# Studies on the preparation of a sufficient carrier from egg protein and carrageenan for cellulase with optimization and application

**DOI:** 10.1038/s41598-025-88092-3

**Published:** 2025-01-31

**Authors:** Marwa I. Wahba, Shireen A. A. Saleh, Walaa A. Abdel Wahab, Faten A. Mostafa

**Affiliations:** 1 Chemistry of Natural and Microbial Products Department, Pharmaceutical Industries Research Institute, National Research Centre, El-Behooth St., Dokki, Giza, 12622 Egypt; 2 Centre of Scientific Excellence-Group of Advanced Materials and Nanotechnology, National Research Centre, El-Behooth St., Dokki, Giza, Egypt

**Keywords:** Biochemistry, Biological techniques

## Abstract

Egg protein (EP) concentration, pH, and glutaraldehyde (GA) concentration were optimized using Box Behnken design (BBD) to prepare GA-EP-Carr (Carrageenan) beads as a carrier for *Aspergillus niger* MK981235 cellulase. It was recommended that the concentrations of GA, and EP be set at 11.21% (w/v), 8% (w/w), and pH 3, respectively. It was determined that 60 °C and 2% for free form and 60 °C and 3% for im-cellulase were the optimum temperature and CMC concentration parameters for maximum enzyme activity. Free and im-cellulase were determined to have K_m_ and V_max_ of 2.22 mg.ml^-1^ and 1.76 µmol.ml^-1^.min^-1^, and 4.55 mg.ml^-1^ and 3.33 µmol.ml^-1^.min^-1^, respectively. Covalent coupling of *A. niger* cellulase to GA- EP- Carr beads improved its thermodynamic parameters T_1/2_ and D-values by 2.48, 2.01, and 2.36 times at 40, 50, and 60 °C, respectively. GA- EP- Carr im-cellulase was 100% active for 60 days at 4 °C and can be used for CMC hydrolysis for 20 successive cycles. GA- EP- Carr im-cellulase showed remarkable efficiency in the clarification of mango, peach, grape, and orange juices emphasized by TSS (total soluble solids), turbidity, and reducing sugar measurements for 3 successive cycles. GA- EP- Carr im-cellulase can be applied with high efficiency in juice industry.

Enzymatic technology is now used more frequently in industrial processes as a result of the present demand for sustainable green techniques. However, the harsh circumstances of industrial operations increase the likelihood of enzyme destabilization, limiting their useful life, as well as the fact that free enzymes typically have poor stability against pH, heat, or other variables and cannot be recovered and used again^1^. Other drawbacks include the high expense and lack of long-term operational stability of utilizing enzymes in large-scale productions. They are challenging to remove from the reaction system, which restricts the recovery of the enzyme and could contaminate the finished product^2^. Therefore, there is a huge need to figure out how to increase the stability and reusability of enzymes. Enzyme immobilization may provide for all of these requirements. The immobilization process has been used extensively as a very effective tool to create a variety of high-performance and financially viable biocatalysts with enhanced stability and reusability for biotechnological applications^3,4^. The immobilized enzymes exhibit greater thermal stability than the free enzymes and can operate over a wider pH and temperature range^5^.

Egg white comprises 88% water and 10.5% protein. Egg white proteins (EPs) comprise variable proteins whose molecular weights and iso-electric points are much varied. Nonetheless, ovalabumin is the chief EP as it constitutes 54% of its composition. Ovalbumin is a glycoprotein with a 45 kDa molecular weight^6^ and a 4.5–4.7 iso-electric point^6,7^. EPs present numerous advantageous functional qualities, such as the foaming qualities which are exploited during the preparation of cakes, meringues, and marshmallows. EP also served as the foaming agent during the foam mat drying of variable vegetable and fruit extracts^6^. Furthermore, EPs are competent emulsifiers^8^ and gelling agents. The gelling qualities of EPs have been exploited during the preparation of food products, such as surimi. These gelling qualities have also enabled the preparation of EP-based nanofibers, nanoparticles, and hydrogels which could be utilized as delivery vectors for variable bioactive species^6^.

EP was also previously utilized to concoct competent covalent immobilizers^9^. Proteins and polysaccharides interact together via electro-static interactions, hydrogen bonding, and also hydrophobic interactions^10^. Thus, EP competently interacted with the beads of the anionic polysaccharide, gellan gum, and formed a dense shell around such beads. The amino-rich EP shell was then exploited to bind glutaraldehyde (GA). GA served to covalently bind enzymes. Moreover, the amino species in the EP shell also served to bind enzymes via ionic-exchange. The obtained GA-EP-gellan gum covalent immobilizers were competent as they offered their immobilized enzyme fine operational, storage, and thermal stabilities^9^. Noteworthy, the operational stability offered by the GA-EP-gellan gum immobilizers was finer than that offered to the selfsame enzyme by the gellan gum immobilizers which were grafted via GA and the commonly utilized, synthetic polyamine; polyethyleneimine (PEI). The GA-EP-gellan gum bound β-galactosidase offered 89.47% activity through its 24th cycle whereas the GA-PEI-gellan gum bound enzyme offered only 81.22% activity through its 14th cycle (last inspected cycle)^9,11^. The competency of the GA-EP-gellan gum immobilizers encouraged the fabrication of other EP based covalent immobilizers.

κ-Carrageenan (Carr) is a biocompatible, biodegradable^10^, anionic polysaccharide which is procured from specific red seaweeds. such as *Kappaphycus alvarezii*,* Hypnea musciformis*, and *Palisada perforata*. Carr is a critical contributor to food industry. However, it lacks the functional species required to covalently link enzymes^12^. This limitation could be overcome if Carr was grafted via EP and GA. Thus, in the current research, Carr was grafted via GA and EP in order to procure novel competent covalent immobilizers. Noteworthy, Carr and EP were formerly investigated together. For instance, EP-Carr films were fabricated in order to serve as green food packaging materials^13^. Moreover, Carr-ovalbumin nano-particles were investigated as delivery vehicles for curcumin^10^. Nonetheless, no EP-Carr covalent immobilizers were formerly fabricated.

Cellulases (EC 3.2.1.4) hydrolyze β-1,4 glycosidic bonds in cellulose, and are applicable in multiple fields, particularly in the wood and cellulose-paper industries, the conservation of thermoplastic polymers and plastics, bioconversion of cellulosic materials to organic solvents, fermentation processes, detergents, textiles, laundry, biofuel and the food and feed industries^14–16^.

In the food field, cellulases are manufactured to enhance the sensory quality of mass, facilitate the extraction and clarification of beverages such as wine and juices, and preserve the rheological stability of the final product^17–20^. Cellulases are used for a variety of processes, including the extraction and clarification of fruit and vegetable juices for the creation of nectars and purees, oil extraction from oil seeds, the preparation of animal feed, an improvement in soaking efficiency, uniform water absorption by cereals, the nutritive quality of fermented foods, the rehydrability of dried vegetables and soups, the creation of oligosaccharides as functional food ingredients and low-calorie food substitutes, and biomass conversion^21–23^.

The most constructing factors that restrict the enzyme application field are the temperature, thermal stability, and influence of metal ions and enzyme inhibitors on cellulase enzyme activity. The effect of magnisum, calcium, zinc, manganese, barium, ferrous, copper sulphate, mercuric, cobalt, sodium and lead on cellulase activity was investigated^24–28^.

Therefore, the preparation of a sufficient carrier composed of egg protein and carrageenan for *Aspergillus niger* MK981235 cellulase was proposed in this study. Investigating the physiochemical, kinetic, thermodynamic, storage, and reusability parameters following the covalent crosslinking between GA- EP- Carr beads and cellulase. Employing the GA- EP- Carr im-cellulase in juice (mango, peach, grape, and orange) clarification for successive cycles. Studying the interaction between GA- EP- Carr im-cellulase amount, mango juice volume, and clarification time on the clarification process to achieve the highest hydrolysis efficiency as reducing sugar amount.

## Materials and methods

### Materials

Carr (Gelcarin GP 812^®^) was acquired from PhytoTechnology Laboratories^®^, USA. 50% GA solution (5.6 M), KCl and CMC were purchased from Sigma, Germany. The EP (spray-dried egg whites powder, minimum protein 80%) was purchased from GF Ovodry S.p.A., Italy.

### Methods

#### Preparation of GA-EP-Carr immobilizers

Powdered Carr was added to distilled water at a 2% (w/w) concentration. This aqueous Carr dispersion was stirred in a 70 °C water bath until Carr dissolved. The hot Carr solution was then dripped via a 22 G needle syringe onto 3% (w/v) KCl solution. The prepared Carr beads were left in KCl solution for at least 6 h. Afterward, they were scrupulously washed and were added to the EP suspension. The pH of the EP suspension was either 1 or 3 or 5, and its concentration was 2, 5, or 8% (w/w) depending on the Box Behnken design (BBD) (Table 1). The mixture was mildly rotated for ~ 18 h on a roller stirrer and then the EP-Carr beads were scrupulously washed and were immersed in a GA solution (concentration either 11.21, 33.64, or 56.07% (w/v)) (Table 1). Finally, the GA-EP-Carr beads were scrupulously washed and were left in distilled water in the fridge till cellulase loading. Noteworthy, the BBD was analyzed via the Design Expert 13 statistical software.

#### Cellulase preparation

*Aspergillus niger* MK981235 was used as cellulase producer by solid-state fermentation technique (SSF) utilizing molokhia stems (MS) as substrate as described previously^29^.

#### Cellulase assay

The enzyme activity was determined by measuring the glucose released from the hydrolysis of carboxymethyl cellulose (CMC) after incubating 0.5 ml of the enzyme with 0.5 ml of CMC (pH 5.00, 0.05 M acetate buffer) for 30 min at 50 °C^30^. The released glucose was determined by the method of Neish^31^. One unit of cellulase (U) was defined as the amount of enzyme librating 1 µmol of glucose per min under the assay conditions.

#### Physiochemical, kinetic, and thermodynamic characterization

The optimum conditions to obtain the maximum cellulase activity including temperature and substrate concentration were investigated. The optimum temperature was investigated by incubating the enzyme with 1% CMC at different temperatures (25, 30, 40, 50, 60, and 70 °C) for 30 min followed by estimating glucose released. The reaction mixture containing different CMC concentrations (0.25, 0.50, 0.75, 1.00, 1.50, 2.00, and 3.00%) was incubated for 30 min under optimum assay conditions. The thermal stability of GA-EP-Carr im-cellulase and free form was investigated by incubating the im-cellulase and free form at 40, 50, and 60 °C for different intervals (15, 30, 45, and 60 min) after which the reaction was carried out under optimum assay conditions. Ca^2+^, Cu^2+^, Fe^3+^, Hg^2+^, K^+^, Mg^2+^, Mn^2+^, Na^+^, Zn^2+^ and Co^2+^ effect on free and im-cellulase were studied at 0.05 M concentration concerning the control without metals as 100%.

The kinetics and thermodynamics of free and GA-EP-Carr im-cellulases were determined as described previously^29^. The kinetics including Michael’s constant (K_m_) and maximum velocity (V_max_) were determined from the Lineweaver-Burk plot. Arrhenius plot of the logarithm of a reaction rate constant plotted against the reciprocal of the temperature and of log denaturation rate constants (ln K_d_) versus the reciprocal of the absolute temperature (K) was employed to determine the activation energy (E_a_) and the activation energy (E_d_), respectively using the equation:

$${\text{Slope}}= - {{\text{E}}_{\text{d}}}/{\text{R}}$$

The following equations were used to determine the denaturation parameters for both free and GA-EP-Carr im-cellulase:

$${{\text{T}}_{{\text{1}}/{\text{2}}}}\left( {{\text{half-life}}} \right)={\text{ln 2}}/{{\text{K}}_{\text{d}}}$$

$${\text{D-value}}\left( {{\text{decimal}}\;{\text{reduction}}\;{\text{time}}} \right)={\text{ln}}\,{\text{1}}0/{{\text{K}}_{\text{d}}}$$

$${\text{D}}{{\text{H}}_{\text{d}}}\left( {{\text{enthalpy}}} \right)={{\text{E}}_{\text{d}}} - {\text{RT}}$$

$${\text{D}}{{\text{G}}_{\text{d}}}\left( {{\text{Gibbs}}\;{\text{native}}\;{\text{energy}}} \right)= - {\text{RT}} \cdot {\text{ln}}({{\text{K}}_{\text{d}}} \cdot {\text{h}}/{{\text{K}}_{\text{b}}} \cdot {\text{T}})$$

where T is the corresponding absolute temperature (K), R is the gas constant (8.314 J mol^−1^ K^−1^), h is the Planck constant (6.626 × 10^−34^ J min), K_b_ is the Boltzman constant (1.38 × 10^−23^ J K^−1^) and K_d_ is the deactivation rate constant (min^−1^).

#### Storage and operational stability

The activity of GA-EP-Carr im-cellulase was assayed (under optimum assay conditions) at different intervals (7, 10, 15, 30, 45, and 60 days) after storage at 4 °C in comparison with free cellulase.

GA-EP-Carr im-cellulase was recycled for repetitive reaction cycles by washing the GA-EP-Carr im-cellulase beads at the end of each reaction with 0.05 M acetate buffer (pH 5.00) and restarting a new reaction under optimum assay conditions (incubating GA-EP-Carr im-cellulase with 0.5 ml of CMC (pH 5.00, 0.05 M acetate buffer) for 30 min at 60 °C and the released glucose was determined by the method of Neish^31^) with the same beads to investigate the operational stability.

#### Juice clarification

Mango, peach, grape, and orange fruits were bought at the open market in Egypt city. The fruits were cut in half, thoroughly cleaned under running water, and then processed in a home juicer. To destroy any naturally occurring fruit enzymes or microorganisms, the pulp was pasteurized at 85 °C for 5 min in a water bath. After cooling to 40 °C, enzyme extract was then added.

After pulp extraction, the enzyme was added to the juice and the efficiency of the clarification process was reflected by using a bench ABBE refractometer, model WY1A, to measure the total soluble solids (TSS, expressed in degrees °Brix), which were measured at a temperature of 30 °C. After clarity, the juice’s absorbance was measured using a spectrophotometer at 600 nm. The reducing sugar content was determined by the method of Neish^31^.

#### Optimization of clarification conditions via CCD

The amount of GA-EP-Carr im-cellulases (g) as factor A, mango juice volume (ml) as factor B, and time of clarification (min) as factor C for the maximum clarification efficiency expressed as reducing sugar (mg) were investigated each with five levels (-1.682, -1.00, 0, 1.00, and 1.682) giving 20 runs. The design was statistically analyzed via ANOVA. Design-Expert^®^8 software from Stat-Ease, Inc. was utilized for experimental design, variance analysis, and process optimization.

### Statistical analysis

Each experiment was the mean of three replicates ± SD (standard deviation of the mean). The data was analyzed by using Microsoft Excel 2019.

## Results and discussion

### BBD

$$\begin{gathered} \operatorname{Im} {\text{-cellulase}}=5.420+0.124{\text{A}}-0.223{\text{B}}-0.286{\text{C}}+0.393{\text{AB}} \hfill \\ \quad \quad \quad \quad \quad \quad \quad -0.707{\text{AC}}-0.045{\text{BC}}-0.095{{\text{A}}^2}-0.543{{\text{B}}^2}+0.618{{\text{C}}^2} \hfill \\ \end{gathered}$$

The optimal EP concentration, EP pH, and GA concentration were pursued via the statistical BBD. Noteworthy, the factors levels adopted during the BBD inspection were determined based on former research. For instance, Carr was formerly optimally grafted with pea-protein and soy-protein-isolate at pHs 1 and 5, respectively^32,33^. Moreover, EP was recommended to graft gellan gum at the pH range 2–4^9^. Thus, we inspected the EP pH in the 1–5 range (Table 1). It was also reported that Carr was optimally grafted with 2.36% (w/w) whey-protein isolate^34^, and that 7% (w/w) EP was the optimal for gellan gum grafting^9^. Accordingly, the inspected EP concentration range was 2–8%, w/w (Table 1). As regards to the GA concentration, 25% (v/v) GA concentration was adopted during Carr grafting with whey-protein-isolate, soy-protein-isolate, and pea protein^32–34^. Thus, a 11.21–56.07% (w/v) GA concentration range was adopted in order to encompass the formerly recommended GA concentration at a near central location.

**Fig. 1:**
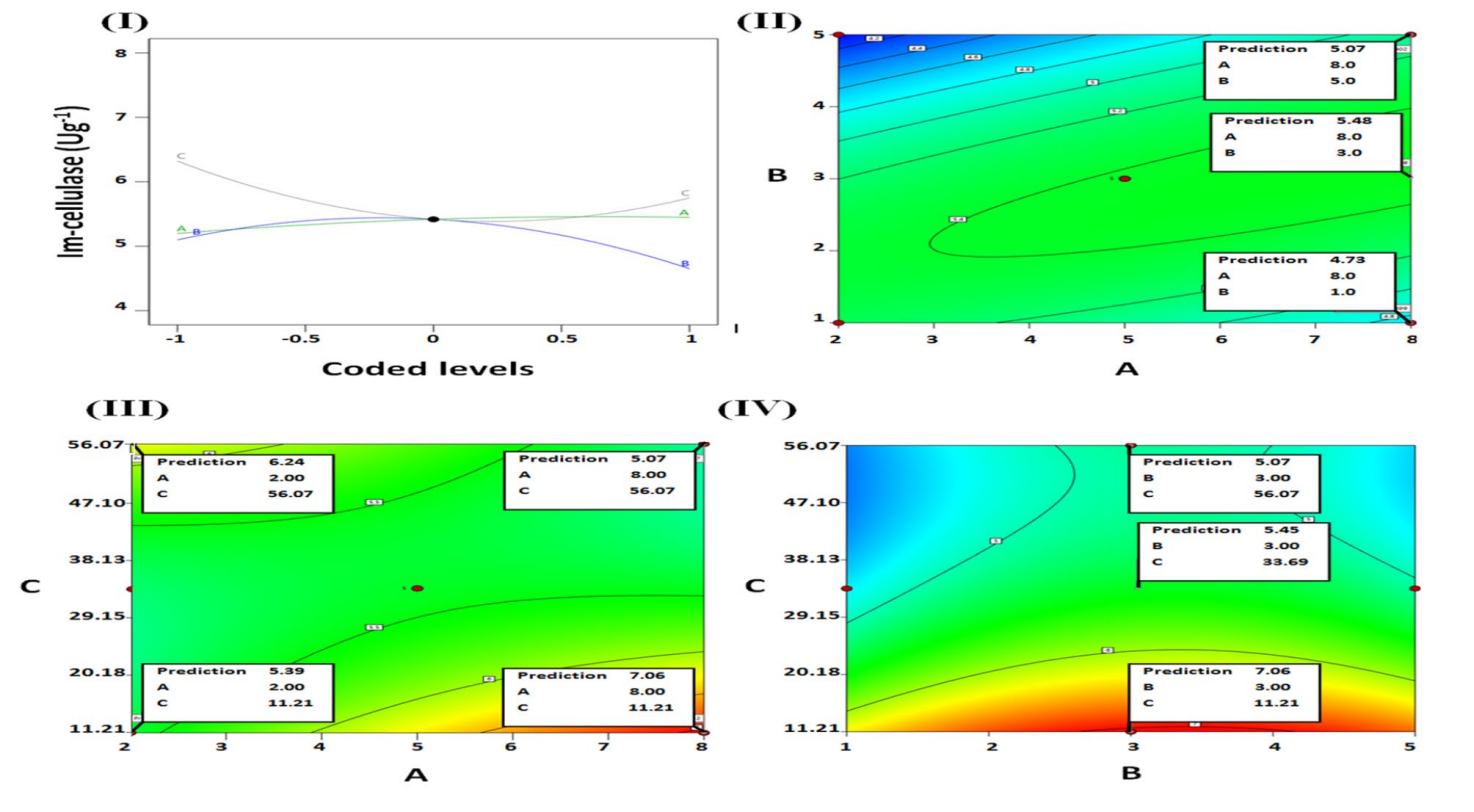
Perturbation plot showing the interaction between the EP concentration (**A**), EP pH (**B**) and GA concentration (**C**).

The analysis of the data retrieved from the BBD (Table 1) revealed that the linear, 2 factor interactions, and quadratic models offered R^2^ values of 0.16, 0.52, and 0.89, respectively. Thus, the quadratic model was chosen owing to its incremented R^2^ which reflected its capability to interpret 89% of the alterations observed within the results. The quadratic model significance was evident from its 0.0107 p-value (Table 2). As regards to the model terms, the glutaraldehyde concentration linear (C) and quadratic (C^2^) terms were significant (p-values 0.0453 and 0.0066, respectively). The EP pH quadratic term (B^2^) was also significant (p-value = 0.0123). On the other hand, the terms representing the EP concentration, whether the linear (A) or the quadratic (A^2^), were insignificant (Table 2). Similarly, the EP concentration was the least influential factor during the GA-EP-gellan gum grafting scheme. It was observed that varying the EP concentration evoked only ~ 1.35 -fold variation in the GA-EP-gellan gum immobilized activity whereas ~ 1.90 and 4.10 -fold variations were evoked by varying the EP pH and GA concentration, respectively^9^. It should also be noted that soy protein isolate concentration was the least influential factor during the GA-soy protein isolate-Carr grafting scheme^32^. Proteins possess bountiful quantities of amino species. These amino species are critical as they are responsible for the binding of the GA species which would mediate the enzymes covalent binding. Amino species could also bind enzymes directly via ionic-exchange^35^. Thus, it could be deduced that the bountiful presence of these critical amino species would enable the GA-protein grafted immobilizers to efficiently bind enzymes even if the protein was adopted at a low concentration. Upon raising the protein concentration, more immobilizer-enzyme interactions would be established and more enzymes would get bound. Nonetheless, elevating the quantity of bound enzymes would cause these enzymes to get packed closely together and this would evoke protein-protein interactions amongst them. The protein-protein interactions could negatively influence enzyme stability^36^, and this would reduce the quantity of active enzyme species^32^. Thus, raising the protein concentration would initially slightly elevate the immobilized activity, but afterwards, it would reduce the immobilized activity owing to crowdedness. Such activity reduction would discourage any further raise in the protein concentration, and the variations recorded after altering the protein concentration would be limited. Noteworthy, the aforementioned slight elevation then slight reduction in immobilized activity was clearly evident in former research when the protein concentration was inspected via the one-factor at a time technique^9,32^. In the case in hand, the insignificance of EP concentration was confirmed from the ANOVA (Table 2) and also from the perturbation plot (Fig. 1(I)). The EP concentration (A) was presented as a nearly linear, non-steep line whereas distinct curvatures were evident in case of the significant factors (EP pH (B) & GA concentration (C).

Although the EP concentration terms (A & A^2^) were insignificant, its interaction term with the GA concentration (AC) was significant (P value = 0.0038). Thus, EP concentration should be kept within the proposed statistical model. Figure 1(III) revealed the outcomes of the AC interaction. Upon utilizing the lowest EP concentration (2%, w/w), elevating the GA concentration from 11.21% (w/v) to 56.07% (w/v) would raise the im-cellulase from 5.39 to 6.24 Ug^−1^. On the contrary, the im-cellulase would be lessened from 7.06 to 5.07 Ug^−1^ if the GA concentration was analogously raised whilst utilizing the topmost EP concentration (8%, w/w). It should be noted that elevating the GA concentration would increment its polymerization degree^35^. Hence, the 56.07% (w/v) GA solution would comprise more sizeable polymeric GA species than would the 11.21% (w/v) GA solution. Furthermore, it was previously stated that utilizing low protein concentrations would limit protein adsorption and this would consequently reduce the steric hindrance and improve diffusion^37^. Thus, if the 2% (w/w) EP concentration was utilized, loose EP shells with reduced steric hindrance would encircle the Carr beads. The reduced steric hindrance within such shells would allow for the diffusion of the sizeable polymeric GA species. This would permit the competent interaction of these sizeable GA species with the immobilizer and would boost the immobilizer functionality when 56.07% (w/v) GA was used. On the other hand, if the 8% (w/w) EP was utilized, the EP shell would be tightly packed. This would impose steric limitations on the interaction of the sizeable polymeric GA species which might only interact with the sterically accessible amino species on the surface of the EP shell. Accordingly, the functionality of the immobilizer would be reduced and the quantity of im-cellulase would decrease if the 8% EP-Carr beads were grafted with 56.07% (w/v) GA solution.

The recommended settings for the EP concentration, EP pH, and GA concentrations were 8% (w/w), pH 3, and 11.21% (w/v), respectively. Such settings were the settings of run 4 (Table 1), which was the run that acquired the uppermost quantity of im-cellulase. Noteworthy, the 8% (w/w) EP concentration, which was recommended herein for the GA-EP-Carr grafting, was close to the 7% (w/w) EP concentration that was optimal for the GA-EP-gellan gum grafting^9^. Moreover, EP pH 3 was also selected for the GA-EP-gellan gum grafting scheme^9^. However, the 11.21% (w/v) GA concentration, recommended herein, was lower than the 20–25% (v/v) and 25% (v/v) GA concentrations selected for the GA-EP-gellan gum and the GA-soy-protein-isolate-Carr grafting schemes, respectively^9,32^. It was also reported that 25% (v/v) GA solution was used during the GA-whey-protein-isolate-Carr grafting^34^. Such altered optimal GA concentrations could be regarded to the variability amidst the immobilized enzymes especially since the EP concentration and pH (7% (w/w), pH 3, respectively) which were adopted during the GA-EP-gellan gum grafting were very close to the values recommended herein^9^. In the case of the GA-EP-gellan gum, the GA-soy-protein-isolate-Carr, and the GA-whey-protein-isolate-Carr immobilizers, a sizeable enzyme (*Aspegillus oryzae* β-galactosidase; m.w.110 kDa) was immobilized^9,32,34^. On the other hand, the GA-EP-Carr was used herein to immobilize the *Aspergillus niger* cellulase whose molecular weight was reported to be only 13.5 kDa^38^. It should also be noted that elevating the GA concentration would increment its polymerization degree^35^. Thus, the 20% and 25% (v/v) GA solutions would comprise more polymeric GA species than would the 11.21% (w/v) GA solution. These polymeric species would be more sizeable and would preferably interact with sterically accessible amine species on the protein surface. This situation would be favored by the sizeable enzymes, such as *A. oryzae* β-galactosidase, and thus, the lofty (20–25% (v/v)) GA concentrations were selected^9,32^. On the other hand, the smaller size of the *Aspergillus niger* cellulase would reduce the steric limitations imposed on its interactions with the GA-EP-Carr. Thus, it wouldn’t require the polymeric GA species to efficiently sterically interact with the immobilizer. This caused the lesser 11.21% (w/v) GA concentration, which would present more monomeric GA species, to be selected. Noteworthy, elevating the GA concentration from the recommended 11.21–33.69% and then to 56.07% (w/v) would lessen the im-cellulase from 7.06 to 5.45 and then to 5.07 Ug^−1^ if 8% (w/w) EP of pH 3 was used (Fig. 1(IV)).

Electrostatic interactions are pivotal contributors to the polysaccharides-proteins interactions^7^. Thus, inspecting the charges presented by EP and Carr could rationalize the selection of the EP pH 3. EP was reported to present a zeta-potential of around 20 mV at pH 3^8^. On the other hand, Carr, which comprises anionic sulfate species (pKa around pH 2), was shown to be anionic amidst 1–7 pH range^39^. Thus, competent electrostatic interactions would be created amidst the cationic EP and the anionic Carr at pH 3. This would allow for the building up of a dense EP shell around the Carr beads and would lead to the procurement of competent GA-EP-Carr immobilizers. Noteworthy, pH 3 was formerly reported to be the optimal pH for the concoction of co-acervates amidst Carr and EP chief protein; ovalbumin^7,10^. Inspecting the co-acervation amidst Carr and ovalbumin also revealed that lowering the pH from 3 to 1.6 or raising it to 5 negatively influenced the Carr-ovalbumin co-acervation^7^. Similarly, in the case in hand, lowering the EP pH from the recommended pH 3 to 1 or raising it to 5 would negatively influence the GA-EP-Carr grafting scheme and would lessen the quantity of im-cellulase from 5.45 to 4.73 and 5.07 Ug^−1^, respectively (Fig. 1(II)). EP iso-electric point is amidst 4.5–5.1^6,8^. Thus, at pH 5, EP net charge would be nearly zero. This would lessen the EP cationic qualities and would negatively influence its electrostatic interaction with the anionic Carr. Thus, less competent GA-EP-Carr immobilizers would be procured and less im-cellulase activity would be procured at pH 5.

As regards the lower im-cellulase activity, which would be procured at pH 1 (Fig. 1(II)), Carr anionic qualities were shown to decline progressively as its pH was reduced from 3 to 1.6^7^ or to 1^29^. The reduced Carr anionic qualities might negatively influence its electros-static interaction with EP at pH 1. Moreover, ovalabumin, which is EP chief constituent and the determinant of EP iso-electric point^6^, was shown to present progressively increased cationic qualities as its pH was reduced from 3 to 1.6^7^. Thus, it could be inferred that EP cationic qualities would also be increased as its pH was reduced from 3 to 1. The EP excessive cationic qualities at pH 1 would induce pronounced electrostatic repulsions amongst its moieties. Noteworthy, elevating the repulsions amongst certain moieties would impede their aggregation^13^. Impeding the aggregation of EP moieties at pH 1 would prohibit the building up of dense EP shell around the Carr beads. This would lead to the procurement of less competent GA-EP-Carr immobilizers. On another occasion, it was argued that the pronounced repulsions that existed amongst EP moieties at pH 1 impeded EP aggregation and led to the creation of an inefficient EP shell amidst gellan-gum beads. This inefficient EP shell didn’t provide sufficient functionalities for competent enzyme immobilization. Moreover, it failed to keep the integrity of the gellan-gum beads and these beads experienced swelling after their overnight soaked within 0.3 M buffer^9^.

**Table 1:** Box Behnken design (BBD) to optimize the the egg protein (EP) concentration, EP pH, and glutaraldehyde (GA) concentration for the preparation of sufficient GA- EP- Carr (Carrageenan) beads.

Run	A: EP concentration (%,w/w)	B: EP pH	C: GA concentration (%, w/v)	Im-cellulase activity (Ug^−1^)
1	8 (+ 1)	3 (0)	56.07 (+ 1)	4.83
2	5 (0)	5 (+ 1)	11.21 (-1)	5.38
3	5 (0)	1 (-1)	11.21 (-1)	5.96
4	8 (+ 1)	3 (0)	11.21 (-1)	7.03
5	2 (-1)	5 (+ 1)	33.64 (0)	4.02
6	5 (0)	3 (0)	33.64 (0)	5.58
7	5 (0)	1 (-1)	56.07 (+ 1)	5.69
8	2 (-1)	3 (0)	11.21 (-1)	5.63
9	2 (-1)	1 (-1)	33.64 (0)	5.03
10	5 (0)	3 (0)	33.64 (0)	5.73
11	5 (0)	3 (0)	33.64 (0)	4.98
12	8 (+ 1)	1 (-1)	33.64 (0)	4.76
13	8 (+ 1)	5 (+ 1)	33.64 (0)	5.31
14	5 (0)	5 (+ 1)	56.07 (+ 1)	4.94
15	5 (0)	3 (0)	33.64 (0)	5.14
16	5 (0)	3 (0)	33.64 (0)	5.66
17	2 (-1)	3 (0)	56.07 (+ 1)	6.26

**Table 2:** Statistical analysis (ANOVA) for BBD.

Source	SS^a^	DF^b^	MS^c^	F-value	*p*-value
Model	6.540	9	0.727	6.570	0.0107
A-EP concentration	0.124	1	0.124	1.120	0.3255
B-EP pH	0.399	1	0.399	3.600	0.0995
C-GA concentration	0.654	1	0.654	5.910	0.0453
AB	0.617	1	0.617	5.570	0.0503
AC	2.000	1	2.000	18.080	0.0038
BC	0.008	1	0.008	0.074	0.7941
A^2^	0.038	1	0.038	0.346	0.5751
B^2^	1.240	1	1.240	11.200	0.0123
C^2^	1.610	1	1.610	14.520	0.0066
Residual	0.775	7	0.111		
Lack of Fit	0.326	3	0.109	0.969	0.4898
Pure Error	0.449	4	0.112		
Cor Total	7.320	16			

^a^Sum of squares.

^b^Degrees of freedom.

^c^Mean square.

### FTIR inspection

**Fig. 2:**
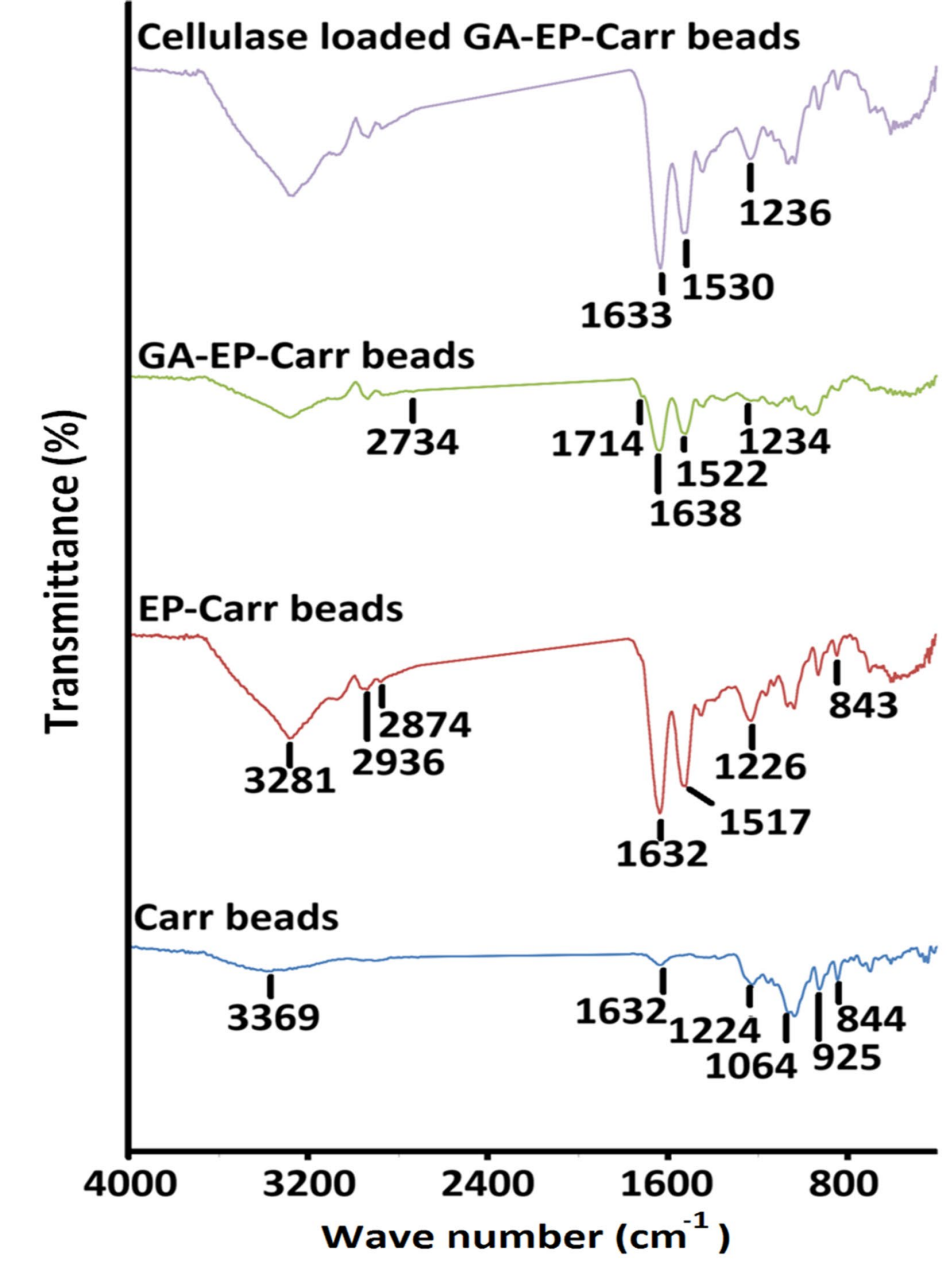
FTIR spectra of plain Carr beads, optimal EP-Carr beads, optimal GA-EP-Carr beads and their cellulase-loaded compeer.

Carr construction comprises ample hydroxyl residues^39^. The H-bonded stretching vibrations of these hydroxyl residues were presented as a broad band at 3369 cm^−1 40^. Figure 2 also revealed the characteristic Carr fingerprint bands which represented the sulphate ester (1224 cm^−1^), the glycosidic bond (1064 cm^−1^), the D-3,6-anhydrogalactose (925 cm^−1^) and the D-4-sulfate-galactose (844 cm^−1^). Noteworthy, the above-mentioned Carr fingerprint bands were reported earlier at 1255 – 1226, 1071 –1064, 928–925, and 847 –843 cm^−1^, respectively^33,40^. The Carr spectrum also presented a band at 1632 cm^−1^ (Fig. 2). Similarly, Chitra et al. (2020) reported a band at 1635 cm^−1^ in i-carrageenan spectrum, and they regarded it to water deformation^41^.

Protein-polysaccharide interactions were formerly stated to involve electro-static interactions, hydrogen bonding, and hydrophobic interactions^10^. The establishment of electro-static interactions and hydrogen bonding amidst EP and Carr could be verified from the EP-Carr spectrum. The EP-Carr spectrum revealed that EP inclusion incremented the area of the OH stretching band and also altered its position to 3281 cm^−1^ (Fig. 2). Noteworthy, Hong et al. (2021) stated that variations in the OH stretching bands could reflect variations in hydrogen-bond networks. Thus, it could be deduced that the EP-Carr interactions involved hydrogen bonding^42^. Likewise, the establishment of hydrogen bonds amidst Carr and pea protein, and also amidst gum tragacanth-agar and polyethyleneimine was deduced from the variations observed in the OH stretching bands^33^. Furthermore, EP inclusion incremented the area of the Carr sulphate ester band and slightly altered its position to 1226 cm^−1^ (Fig. 2). The D-4-sulfate-galactose was also slightly shifted to 843 cm^−1^. These alterations could be regarded to the alterations evoked in the Carr sulphate residues following their interaction with EP. Thus, electrostatic interactions were also involved in the EP-Carr interactions. Noteworthy, the above-mentioned Carr sulphate ester band alterations could have also been evoked by the arousal of EP amide III band (1238 –1230 cm^−1^)^40^, which represents the C-NH_2_ stretching vibration^7^. EP amide I band (1635 –1630 cm^−1^)^40^; C = O stretching vibration^7^ also overlapped with the 1632 cm^−1^ Carr band, and this significantly incremented the area of the 1632 cm^−1^ band in the EP-Carr spectrum. As regards to the EP amide II band (1526 –1517 cm^−1 40^; C-N stretching and N-H bending vibrations^7^, it appeared as a new band at 1522 cm^−1^. Figure 2 also unveiled two new bands in the EP-Carr spectrum at 2936 and 2874 cm^−1^. The elaboration of these two bands further verified the merger of EP as EP FTIR spectrum was formerly shown to present a band at 2930 cm^−1^ and another band at a somewhat lower wave-number which was proximate to our 2874 cm^−1^ recorded band^43^.

The GA aldehyde moieties should be presented in the GA-EP-Carr spectrum. Aldehydes are presented in FTIR by the 2800 –2700 cm^−1^ and the 1700–1750 cm^−1^ bands^44^. In the case in hand, a slight indentation and a shoulder newly appeared at 2734 cm^−1^ and 1714 cm^−1^, respectively, in the GA-EP-Carr spectrum. Thus, they could be regarded to GA aldehyde moieties. Likewise, the shoulder that appeared at 1719 cm^−1^ in the GA-pea protein-Carr spectrum was regarded to the GA aldehyde moieties^33^. Noteworthy, cellulase amide I, II, and III bands were evident in the loaded GA-EP-Carr spectrum. The arousal of these bands incremented the area of the 1638, 1522, and 1234 cm^−1^ bands, which were present in the GA-EP-Carr spectrum, and altered their positions to 1633, 1530, and 1236 cm^−1^, respectively.

### Physiochemical characterization

**Fig. 3:**
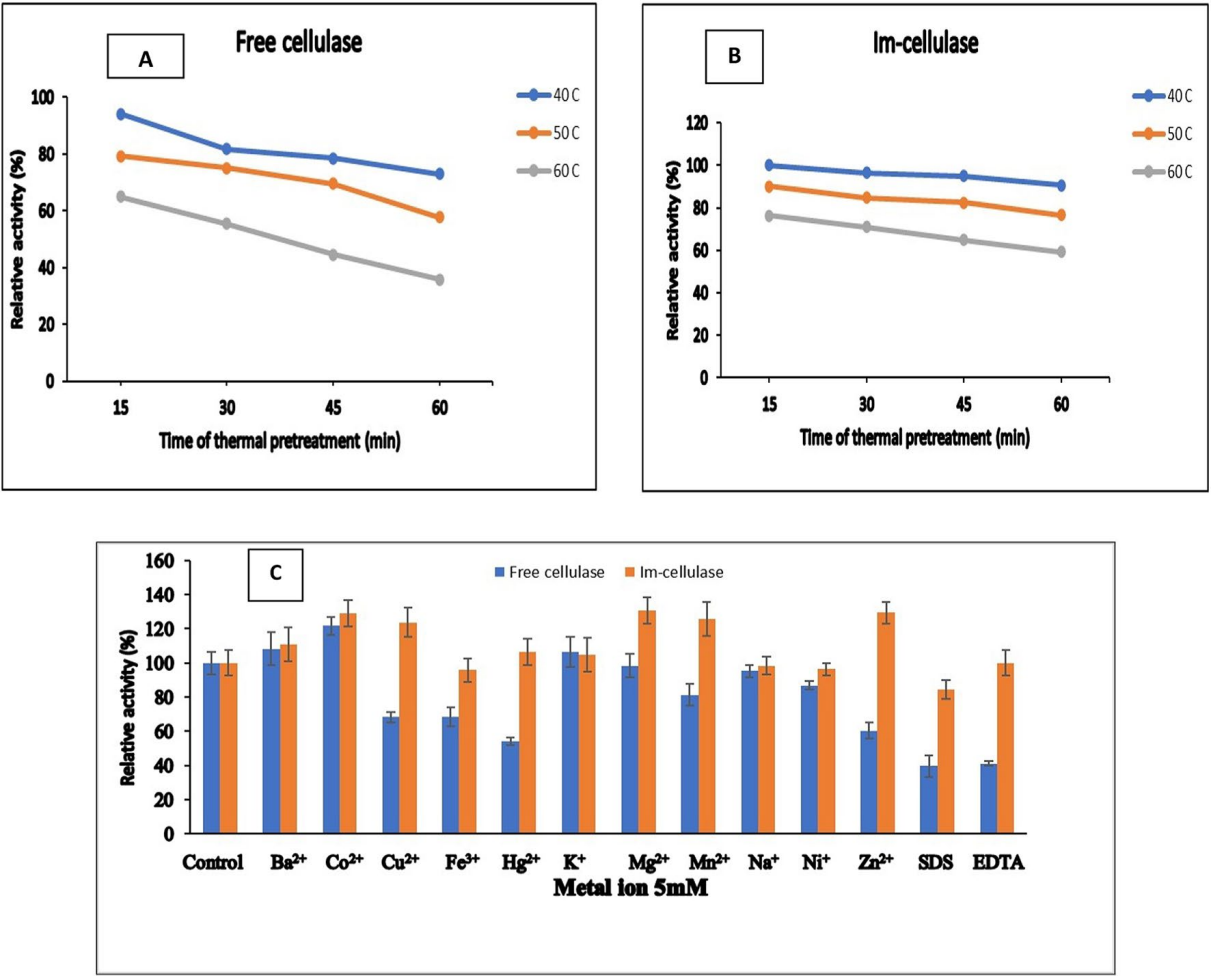
Physiochemical characterization of free and Im-cellulase, (**A**) and (**B**) Thermal stability of free and immobilized forms of *A. niger* cellulase, respectively; (**C**) Effect of metal ions on free and immobilized forms.

Regarding the protein nature of enzymes, they are highly affected by temperature. Both free and im-cellulase showed the highest activity at 60 °C (data not shown). Similar to *A. niger* cellulase, which was deposited on the inorganic–organic hybrid matrix^45^, crosslinked to magnetic nanoparticles^46^, and cellulase from termites trapped on calcium alginate beads^47^, higher than that reported to cellulase (commercial from Novozymes) immobilized by adsorption and by reticulation^48^ and *A. hortai* cellulase immobilized on poly (Acrylamide-Co-Acrylic Acid)^49^ and *Stenotrophomonas maltophilia* cellulase immobilized on an agar-agarose matrix^50^. But the rise in temperature effect was more pronounced on the free form at 70 °C it lost 28% of its activity while the immobilized form lost 10% under the same conditions. The thermal stability profile of *A. niger* free and im-cellulase (Fig. 3A, B) emphasized how the immobilization process protected the enzyme from thermal denaturation. i.e. im-cellulase lost 9.45, 23.29, and 40.62% compared to 27, 42.27, and 64.7% of their original activity after heat pretreatment for 60 min at 40, 50, and 60 °C, respectively. Zdarta et al. (2017) reported a significant improvement in the thermal stability of *A. niger* cellulase after immobilization on TiO2–lignin.

hybrid that it retained over 75% and 80% of its activity after heat pretreatment at 50 and 60 °C, respectively while the free form lost 80% of its activity^45^. The improvement effect of the immobilization process on the thermal stability of enzymes was also reported by Talekar et al. (2013)^51^. The optimum CMC concentration to obtain the highest activity by free (3.52 U.ml^−1^) and im-cellulase (3.08 U.ml^−1^) were 2 and 3%, respectively. The covalent coupling of *A. niger* cellulase to GA- EP- Carr beads preserved it from the inhibitory effect metals as Cu^2+^ and Hg^2+^ and SDS and EDTA as shown in Fig. 3C. According to Yousef and Mawad (2023) Mg^2+^ ,Cu^2+^, Mn^+ 2^, and Ca^2+^ have inhibitory effect on *Virgibacillus salaries* cellulase activity^27^. Malik and Javed (2021) reported the acceleration effect of Zn^2+^, Mn^2+^, and Mg^2+^ on *Bacillus subtilis* cellulase^28^. Tamilanban et al. (2018) reported the promotive effect of Co^2+^, Na^+^, K^+^, and Zn^2+^ on *Stenotrophomonas maltophilia* cellulase and Mn^2+^ and Cu^2+^ in agar-agarose matrix^50^. Metal ions can bind with proteins and form complexes with other molecules related to enzymes, functioning as electron donors or acceptors as Lewis acids, or as structural regulators^52^. These ions can either activate or inhibit enzyme activity by interacting with the amine or carboxylic acid groups of amino acids^53^.

### Kinetic and thermodynamic characterization

**Fig. 4:**
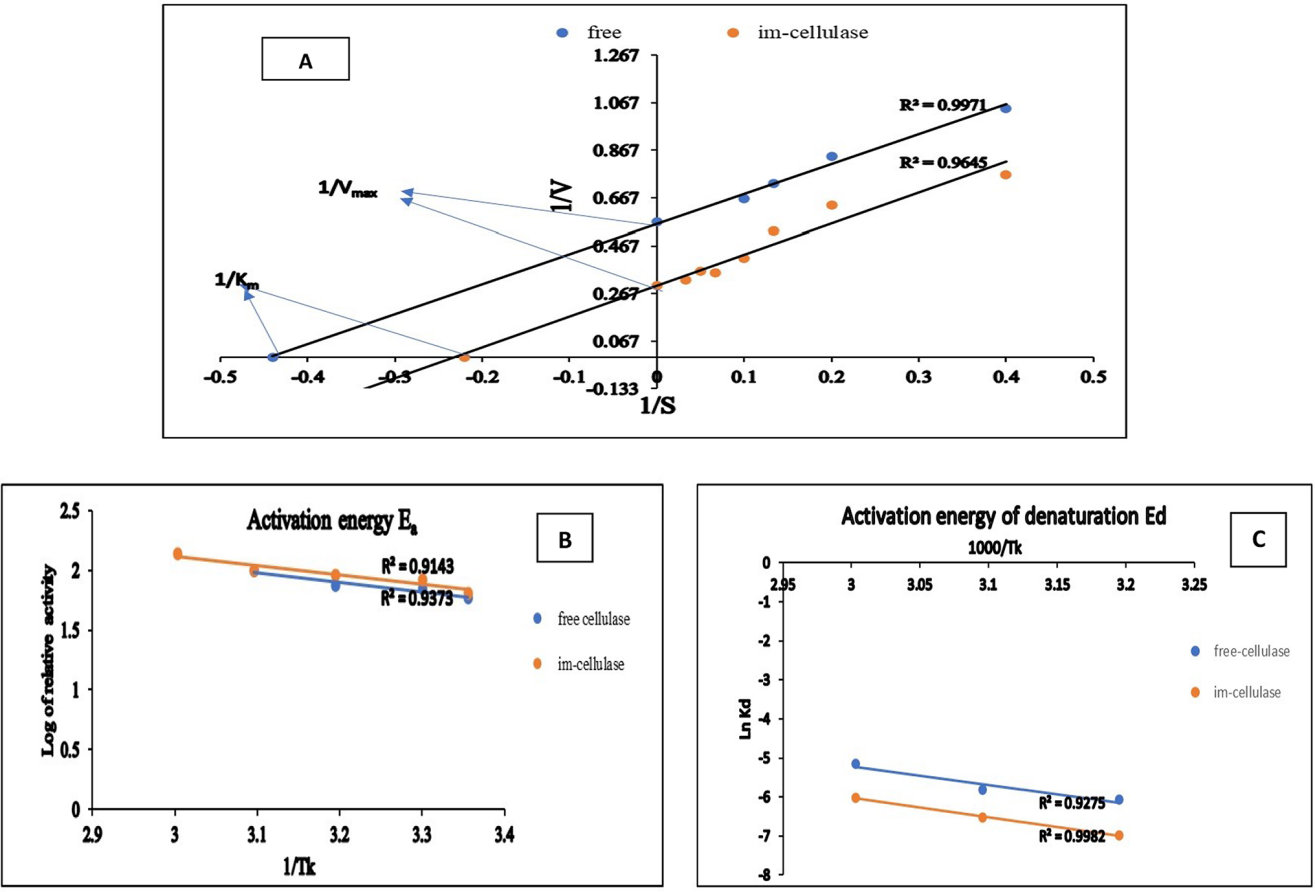
Kinetic and thermodynamic characterization of free and im-cellulase, (**A**) Lineweaver–Burk plot for K_m_ and V_max_ determination; (**B**) and (**C**) Arrhenius plots for E_a_ and E_d_ determination, respectively.

K_m_ and V_max_ for free and im- cellulase (Fig. 4A) as calculated from Linweaver- Burk plot reflecting the enzyme affinity for the substrate were 2.22 mg.ml^-1^ and 1.76 µmol.ml^-1^.min^-1^, respectively for free *A. niger* cellulase and 4.55 mg.ml^-1^ and 3.33 µmol.ml^-1^.min^-1^, respectively for im-cellulase. Romo-Sánchez (2014) reported an increase in K_m_ value for cellulase immobilized by adsorption and reticulation techniques^48^. The immobilization process in some studies as reported by Zdarta et al. (2017)^45^ and Muley et al. (2018)^46^ raised the value of K_m_ and V_max_. The increase in K_m_ value for im-cellulase can be attributed to the more difficulty offered by the immobilization process for the contact between the enzyme and the substrate as confirmed by El-Hadi et al. (2014)^49^.

The amount of energy needed to initiate the reaction, known as the activation energy E_a_, is low because the enzyme is stable, whereas the amount of energy needed to cause denaturation, known as the activation energy of denaturation E_d_, is high because the enzyme is stable. The covalent binding of *A. niger* cellulase onto GA- EP- Carr beads changed the E_a_ and E_d_ values from 15.44 to 39.24 kJ.mol^-1^, respectively for the free form to 12.47 and 41.54 kJ mol^-1^, respectively for im-cellulase as calculated from Arrhenius plots (Fig. 4B, C). The improvement in Ed for im-form of cellulase was reported by Mull et al. (2018)^46^ and Ladole et al. (2017)^54^. This demonstrated unequivocally that, in contrast to the free enzyme, a certain excess of energy was needed to alter the native conformation of the enzyme immobilized onto GA- EP- Carr beads. This could be because the newly created covalent bonds preserve and stabilize the structural conformation of immobilized enzymes^55^.

T_1/2_ and D-values are thermal parameters that declare the duration of time that the enzyme needs to lose 50, and 90% of its activity, respectively. The immobilization of *A. niger* cellulase in this study prolonged this time from 297.92, and 989.68 to 739.57, and 2456.82 min at 40 °C for free and im-cellulase, respectively as shown in Table 3. More parameters as ΔH_d_ (total energy needed to deactivate an enzyme), ΔG_d_ (the quantity of useful energy that is produced when an enzyme deactivates and also evaluates the spontaneity of the thermal inactivation process brought on by conformational distortion and the breaking of multiple bonds).

, ΔS_d_ (the energy per degree required for local disordering between the transition and ground state or for the conversion of native enzymes to denatured forms) were calculated as remarks for the thermal stability of the enzyme. As the value of ΔH_d_, ΔG_d_ increase as the stability increases in contrast to ΔS_d_ value there is a reverse relation between enzyme stability and ΔS_d_. Table 3 reflected many findings, as the temperature increased as the magnitude of ΔH_d_ and ΔG_d_ decreased, reflecting that lesser energy was required for enzyme denaturation at higher temperatures, the destructive effect of temperature on the enzyme is more pronounced on free form than on the im-form emphasizing the protective effect of immobilization process on enzyme^46,56^. The disruption of hydrogen bonds and/or the exposure of hydrophobic regions to the native protein surface from the interior, or vice versa, are conformational changes that occur during enzyme denaturation, which can be attributed to positive ΔH° values at elevated temperatures^57^.

It should also be noted that the GA-EP-Carr beads kept their integrity after their 1 h thermal incubations at 40–60 °C. On the contrary, potassium hardened Carr disks were stated to dissolve following 1 h incubation at 35 °C^58^. This implied the incremented thermal stability of the GA-EP-Carr beads which could be regarded to the GA cross-linked EP shell which encircled the Carr beads.

**Table 3:** Thermodynamic parameters of denaturation for free and im-cellulase.

*P*	k_d_ (min^−1^)		T1/2 (min)		D-value (min)		ΔH_d_ (kJ mol^−1^)		ΔG_d_ (kJ mol^−1^)		ΔS_d_ (J mol K^−1^)	
T	F	Im	F	Im	F	Im	F	Im	F	Im	F	Im
40	0.002327	0.000937	297.9242	739.57	989.68	2456.81	36.64	38.93	92.56	94.92	-178.66	-178.88
50	0.002987	0.001483	232.0597	467.30	770.88	1552.34	36.55	38.85	94.93	96.81	-180.72	-179.43
60	0.005781	0.002446	119.8941	283.33	398.27	941.23	36.47	38.76	96.12	98.50	-179.13	-179.39

P: property, T: temperature, F: free, Im: immobilized.

### Storage and operational stability

The main advantage of the immobilization process is the preservation of enzyme activity enabling its use for extended time. In this study, the free *A. niger* cellulase lost 20, 50, 80% of its original activity after 7, 10, 15 days of storage at 4 °C and became completely inactive after 20 days while the im-cellulase was completely active for 60 days at 4 °C. Cellulase from *Penicillium brevicompactum* immobilized by encapsulation in ca-alginate beads lost 71.44% of its activity after 5 weeks^59^. Cellulases (*A. niger* and *Streptomyces sp. G12*) immobilized on poliacrylic beads remained with full activity for 45 days compared to the free enzymes^60^. Zdarta et al. (2017) emphasized that *A. niger* cellulase immobilized on inorganic–organic hybrid matrix kept over 95% of its activity after storage at 4 °C for 30 days while the free form had less than 75% of its activity under same conditions^45^. Mull et al. (2018) reported the stability of tri-enzyme (cellulase, pectinase, and xylanase) co-immobilized on magnetic nanoparticles for 36 days at 5 °C^46^. Another advantage offered by the immobilization process is the possibility of utilizing the immobilized enzyme several times participating in lowering the cost. Im-cellulase on GA-EP-Carr beads was able to hydrolyze CMC for 20 successive cycles with 100% activity. Romo-Sánchez (2014) found that cellulase immobilized via reticulation maintained up to 64% of its initial activity after 19 cycles, whereas the enzyme immobilized via adsorption only preserved 32% of its initial activity^48^. Zdarta et al. (2017) utilized *A. niger* cellulase immobilized on a TiO2–lignin hybrid support for 10 cycles after which it lost 7% of its activity^45^.

### Application of GA-EP-Carr Im-cellulase in juice clarification

The juice clarifying process in the juice industry is critical to reduce the colloidal particles in suspension. These substances make juice turbid and may make consumers less likely to accept the product^17,23^. Results in Table 4 declared some points as, GA-EP-Carr im-cellulase succeeded in improving the quality of mango, peach, orange, and grape juices. The degree of juice clarification deferred according to the fruit type. The best clarification percent was indicated by an increase in total soluble solids (TSS), a decrease in turbidity (low absorbance at 600), and an increase in reducing sugar as declared by Santana et al. (2021)^17^. The increase in reducing sugar content of papaya juice was reported after the treatment with tri-enzyme (pectinase, cellulase, and xylanase) co-immobilized magnetic complex by Mull et al. (2018)^46^. Ozyilmaz and Gunay (2023) reported an increase in reducing sugar and a decrease in turbidity contents of apple, grape, and pear juices after treatment with amylase, pectinase, and cellulase enzyme co-immobilized onto silica gel^61^. The increase in TSS is correlated with a higher degree of tissue breakdown due to the release of sugars^62^. GA-EP-Carr im-cellulase succeeded in clarifying mango, orange, grape, and peach juices for 3 successive cycles with the highest clarification efficiency on mango juice. The declarification efficiency decreased from cycle 1 to cycle 3 can be attributed to the leakage of the enzyme from the beads. There is no change in juice pH. Cellulase from either plant or microbial sources was utilized to clarify juices^17,18^.

**Table 4:** Clarification of different juices with GA- EP- Carr Im-cellulase.

Property				
Cycle	Mango	Orange	Peach	Grape
TSS (°Brix) control	6.55	6.85	6.90	4.00
1st	7.00	7.00	7.00	4.00
2nd	7.30	7.10	7.05	4.15
3rd	7.55	7.25	7.10	4.25
pH control	4.41	4.40	4.25	4.25
1st	4.41	4.40	4.25	4.25
2nd	4.45	4.40	4.25	4.30
3rd	4.50	4.45	4.30	4.30
Reduction in turbidity (%)				
1st	5.25	3.00	3.00	2.85
2nd	4.89	2.25	2.00	2.00
3rd	3.00	1.00	0.95	0.75
Reducing sugar (mg)				
1st	4.4312	2.9812	3.5264	3.7584
2nd	3.886	2.436	3.4916	3.19
3rd	3.132	2.2968	3.3176	2.1228

### CCD for clarification conditions optima

**Fig. 5:**
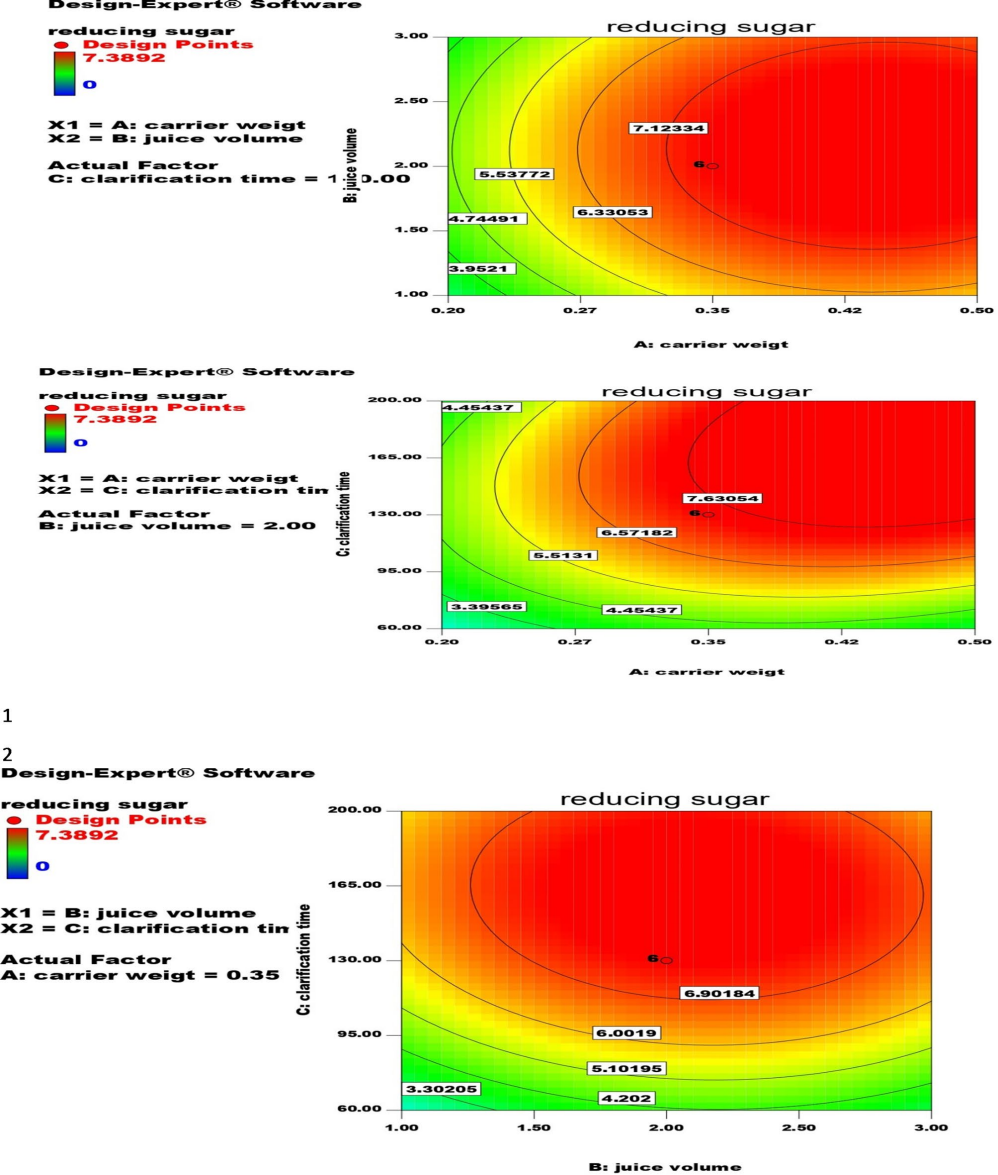
Contour plots that investigated the consequences of modifying the GA- EP- Carr weight (**A**), the juice volume (**B**), and the clarification time (**C**) on the reducing sugar.

The interaction between the amount of GA-EP-Carr im-cellulase (enzyme dose), mango juice volume (ml), and clarification time (min) effect on clarification efficiency represented by reducing sugar amount (mg) was investigated. Santana et al. (2021) investigated the interaction between cellulase dose and clarification time on tangerine juice clarification expressed by reduction in juice viscosity^17^. As shown in Table 5; Fig. 5 the interaction between these factors led to wide variation in reducing sugar content 0-7.39 mg with the highest hydrolysis yield (7.39 mg) in central runs (3,4, 6,8, 15, and 19). The reducing sugar yield (mg) can be calculated from the following equation:

$$\begin{gathered} {\text{Reducing}}\;{\text{sugar}}\;\left( {{\text{mg}}} \right)=+{\text{7}}.{\text{41}}+{\text{1}}.{\text{52}}*{\text{ GA-EP-Carr}}\;{\text{im-cellulase}}+0.{\text{35}}*{\text{mango}}\;{\text{juice}}\;{\text{volume}} \hfill \\ \quad +{\text{1}}.{\text{6}}0*{\text{clarification time}}+0.0{\text{7}}0*{\text{GA-EP-Carr}}\;{\text{im-cellulase}}*{\text{mango}}\;{\text{uice}}\;{\text{volume}} \hfill \\ \quad +0.{\text{94}}*{\text{ GA-EP-Carr}}\;{\text{im-cellulase}}*{\text{clarification}}\;{\text{time}} - \,0.{\text{14}}*{\text{mango}}\;{\text{juice}}\;{\text{volume}}*{\text{clarification}}\;{\text{time}} \hfill \\ \quad - {\text{1}}.{\text{21}}*{\text{GA-EP-Carr}}\;{\text{im-cellulas}}{{\text{e}}^{\text{2}}} - {\text{1}}.{\text{23}}*{\text{mango}}\;{\text{juice}}\;{\text{volum}}{{\text{e}}^{\text{2}}} - {\text{1}}.{\text{68}}*{\text{clarification}}\;{\text{tim}}{{\text{e}}^{\text{2}}} \hfill \\ \end{gathered}$$

The statistical analysis of the design as shown in the Table 6 (ANOVA) and values of R^2^, adj R^2^, and pred R^2^ (0.9771, 0.9565, and 0.8175, respectively) revealed the success of the design. The F-value of the model (47.00) implied the significance of the design. According to the statistical analysis A, C, AC, A2, B2, C2 were proved to be significant model terms since their.

p-value was less than 0.05.

**Table 5:** CCD for optimization of clarification process.

Run	Factor 1 A: carrier weight (g)	Factor 2 B: juice volume (ml)	Factor 3 C: clarification time (min)	Reducing sugar (mg)
1	0.35	0.318	130	3.224
2	0.2	3	200	2
3	0.35	2	130	7.389
4	0.35	2	130	7.389
5	0.5	1	60	1.856
6	0.35	2	130	7.389
7	0.35	2	247.72	5.88
8	0.35	2	130	7.389
9	0.5	1	200	6.368
10	0.2	1	200	2.691
11	0.35	3.682	130	5.17
12	0.35	2	12.27	0
13	0.098	2	130	1.148
14	0.602	2	130	7.389
15	0.35	2	130	7.389
16	0.5	3	200	7.25
17	0.5	3	60	2.007
18	0.2	1	60	0.661
19	0.35	2	130	7.389
20	0.2	3	60	1.821

Each 0.35 g carrier was loaded with 20 U of *A. niger* cellulase.

**Table 6:** Statistical analysis for clarification CCD (ANOVA).

Source	Sum of squares	df	Mean square	F value	*p*-value Prob > F	
Model	146.0044	9	16.22272	47.40104	< 0.0001	significant
A-carrier weight	31.68948	1	31.68948	92.59329	< 0.0001	
B-juice volume	1.667876	1	1.667876	4.873355	0.0518	
C-clarification time	34.96915	1	34.96915	102.1761	< 0.0001	
AB	0.039706	1	0.039706	0.116016	0.7404	
AC	7.119274	1	7.119274	20.80176	0.0010	
BC	0.156912	1	0.156912	0.45848	0.5137	
A^2^	21.07492	1	21.07492	61.57867	< 0.0001	
B^2^	21.96397	1	21.96397	64.17639	< 0.0001	
C^2^	40.63061	1	40.63061	118.7183	< 0.0001	
Residual	3.422438	10	0.342244			
Lack of fit	3.422438	5	0.684488			
Pure error	0	5	0			
Cor total	149.4269	19				

## Conclusion

The covalent binding of *A. niger* MK981235 cellulase onto GA- EP- Carr beads improved its thermal stability as the values E_a_ and E_d_ altered from 15.44 to 39.24 kJ.mol^−1^, respectively to 12.47 and 41.54 kJ mol^−1^. Additionally, the T_1/2_ and D-values increased by 2.48, 2.01, and 2.36 -folds at 40, 50, and 60 °C, respectively. Without experiencing any activity loss, the im-cellulase hydrolyzed CMC for 20 consecutive cycles. It can also maintain its activity for 60 days when stored at 4 °C. The maximum mango juice clarity efficiency, measured in terms of reducing sugar (7.38 mg), was attained after 130 min using 0.35 g of beads infused with 20.09 U of cellulase for 2 ml of juice. The prepared GA- EP- Carr cellulase can be used in the juice industry.

## Data Availability

The datasets used and/or analysed during the current study available from the corresponding author on reasonable request.

## References

[1] Kim, J., Grate, J. W. & Wang, P. Nanostructures for enzyme stabilization. *Chem. Eng. Sci.* **61**(3), 1017–1026. 10.1016/j.ces.2005.05.067 (2006).

[2] Vallet-regi, M., Ramila, A., Del Real, R. P. & Pérez-pariente, J. A new property of MCM-41: Drug delivery system. *Chem. Mater.* **13**(2), 308–311. 10.1021/cm0011559 (2001).

[3] Tischer, W. & Wedekind, F. Immobilized enzymes: methods and applications. *Top. Curr. Chem.* **200**, 96–126 (1999). https://api.semanticscholar.org/CorpusID:28265025

[4] Barbosa, O. et al. Strategies for the one-step immobilization-purification of enzymes as industrial biocatalysts. *Biotechnol. Adv.* **33**, 435–456. 10.1016/j.biotechadv.2015.03.006 (2015). 25777494 10.1016/j.biotechadv.2015.03.006

[5] Xu, J. et al. Application of iron magnetic nanoparticles in protein immobilization. *Molecules* **19**, 11465–11486. 10.3390/molecules190811465 (2014). 25093986 10.3390/molecules190811465 PMC6270831

[6] Razi, S. M., Fahim, H., Amirabadi, S. & Rashidinejad, A. An overview of the functional properties of egg white proteins and their application in the food industry. *Food Hydrocoll.* **135**, 108183. 10.1016/j.foodhyd.2022.108183 (2023).

[7] Lu, Z. et al. Structural transitions of ovalbumin/κ-carrageenan complexes under the effects of pH and composition. *Chem. Phys.* **533**, 110733. 10.1016/j.chemphys.2020.110733 (2020).

[8] Su, Y. et al. Encapsulation and release of egg white protein in alginate microgels: Impact of pH and thermal treatment. *Food Res. Int.* **120**, 305–311. 10.1016/j.foodres.2019.02.048 (2019). 31000243 10.1016/j.foodres.2019.02.048

[9] Wahba, M. I. Mechanically stable egg white protein based immobilization carrier for β-D-galactosidase: Thermodynamics and application in whey lactose hydrolysis. *React. Funct. Polym.* **155**, 104696. 10.1016/j.reactfunctpolym.2020.104696 (2020).

[10] Xie, H. et al. Fabrication of ovalbumin/κ-carrageenan complex nanoparticles as a novel carrier for curcumin delivery. *Food Hydrocoll.* **89**, 111–121. 10.1016/j.foodhyd.2018.10.027 (2019).

[11] Wahba, M. I. Processed gellan gum beads as covalent immobilization carriers. *Biocatal. Agric. Biotechnol.* **14**, 270–278. 10.1016/j.bcab.2018.03.019 (2018).

[12] Khotimchenko, M. et al. Antitumor potential of carrageenans from marine red algae. *Carbohydr. Polym.* **246**, 116569. 10.1016/j.carbpol.2020.116568 (2020). 10.1016/j.carbpol.2020.116568 32747241

[13] Liu, L., Huang, X., Geng, F. & Huang, Q. Optimization of preparation process of egg white protein/κ-carrageenan composite film. *J. Food Process. Preserv.* **46**, e16167. 10.1111/jfpp.16167 (2022).

[14] Cao, L. *Carrier-Bound Immobilized Enzymes: Principles, Application and Design* (Wiley-VCh, 2005). 10.1002/3527607668.

[15] Kuhad, R. C., Gupta, R. & Singh, A. Microbial cellulases and their industrial applications. *Enzyme Res.* **2010**, 280696. 10.4061/2011/280696 (2011). 10.4061/2011/280696 PMC3168787 21912738

[16] Srivastava, N. et al. Applications of fungal cellulases in biofuel production: Advances and limitations. *Renew. Sustain Energy Rev.* **82**, 2379–2386. 10.1016/j.rser.2017.08.074 (2018).

[17] Santana, M. L. et al. Clarification of tangerine juice using cellulases from *Pseudoyma* Sp. *J. Food Sci. Technol.* **58**(1), 44–51. 10.1007/s13197-020-04511-5 (2021). 33505050 10.1007/s13197-020-04511-5 PMC7813899

[18] Singh, A., Gupta, P. & Wadhwa, N. Cellulase from stored *Amorphophallus paeoniifolius* in clarification of apple juice. *Int. Food Res. J.* **22**(2), 840–843 (2015).

[19] Wang, F. et al. Application of immobilized enzymes in juice clarification. *Foods* **12**, 4258. 10.3390/foods12234258 (2023). 38231709 10.3390/foods12234258 PMC10706659

[20] Dal Magro, L. et al. Magnetic biocatalysts of pectinase and cellulase: Synthesis and characterization of two preparations for application in grape juice clarification. *Int. J. Biol. Macromol.* **115**, 35–44. 10.1016/j.ijbiomac.2018.04.028 (2018). 29634966 10.1016/j.ijbiomac.2018.04.028

[21] Beguin, P. & Aubert, J. P. The biological degradation of cellulose. *FEMS Microbiol. Rev.* **13**, 25–58. 10.1016/0168-6445(94)90099-X (1994). 8117466 10.1111/j.1574-6976.1994.tb00033.x

[22] Bhat, M. K. & Bhat, S. Cellulose degrading enzymes and their potential industrial applications. *Biotechnol. Adv.* **15**, 583–620. 10.1016/S0734-9750(97)00006-2 (1997). 14538158 10.1016/s0734-9750(97)00006-2

[23] Sharma, H. P., Patel, H. & Sugandha. Enzymatic added extraction and clarification of fruit juices–A review. *Crit. Rev. Food Sci. Nut* **57**(6), 1215–1227. 10.1080/10408398.2014.977434 (2017). 10.1080/10408398.2014.977434 26731188

[24] Okonkwo, I. F. Effect of metal ions and enzyme inhibitor on the activity of cellulase enzyme of *Aspergillus flavus*. *IJEAB* **4**(3), 727–734. 10.22161/ijeab/4.3.20 (2019).

[25] Pereira, J. D. C. et al. Effect of metal ions, chemical agents and organic compounds on lignocellulolytic enzymes activities. *Enzyme Inhib. Activ.*. 10.5772/65934 (2017).

[26] Onyia, D. C. et al. The influence of metal ions on cellulolytic activities of fungal isolates from palm biomass. *Nig. J. Biotechnol.* **38**(2), 67–72. 10.4314/njb.v38i2.7 (2021).

[27] Yousef, N. M. H. & Mawad, A. M. M. Characterization of thermo/halo stable cellulase produced from halophilic *Virgibacillus salarius* BM–02 using non–pretreated biomass. *World J. Microbiol. Biotechnol.* **39**, 22. 10.1007/s11274-022-03446-7 (2023). 10.1007/s11274-022-03446-7 PMC9691493 36422734

[28] Malik, W. A. & Javed, S. Biochemical characterization of cellulase from *Bacillus subtilis* strain and its effect on digestibility and structural modifications of lignocellulose rich biomass. *Front. Bioeng. Biotechnol.* **9**, 15. 10.3389/fbioe.2021.800265 (2021). 800265. 10.3389/fbioe.2021.800265 PMC8721162 34988069

[29] Saleh, S. A. A. et al. Characterization of *Aspergillus niger* MK981235 xylanase with extraction of anti-hepatotoxic, antioxidant, hypocholesterolemic and prebiotic *Corchorus olitorius* stems xylooligosaccharides. *Int. J. Biol. Macromol.* **166**, 677–686. 10.1016/j.ijbiomac.2020.10.225 (2021). 33152359 10.1016/j.ijbiomac.2020.10.225

[30] Mandels, M. & Weber, J. The production of cellulases. *Adv. Chem. Ser.* **95**, 391–414. 10.1021/ba-1969-0095.ch023 (1969).

[31] Neish, N. A. Analytical methods for bacterial fermentation, Report no., 46-8-3, National Research Council of Canada, Second Revision. P.34. 10.4224/21273361 (1952).

[32] Wahba, M. I. Soy protein isolate for enzymes bio-conjugation. *Biocatal. Agric. Biotechnol.* **43**, 102390. 10.1016/j.bcab.2022.102390 (2022).

[33] Wahba, M. I. Glutaraldehyde-pea protein grafted polysaccharide matrices for functioning as covalent immobilizers. *Sci. Rep.* **13**, 9105. 10.1038/s41598-023-36045-z (2023). 37277367 10.1038/s41598-023-36045-z PMC10241775

[34] Wahba, M. I. & Soliman, T. N. Whey protein isolate for the preparation of covalent immobilization beads. *Biocatal. Agric. Biotechnol.* **14**, 328–337. 10.1016/j.bcab.2018.04.003 (2018).

[35] Barbosa, O. et al. Glutaraldehyde in bio-catalysts design: A useful crosslinker and a versatile tool in enzyme immobilization. *RSC Adv.* **4**, 583–1600. 10.1039/C3RA45991H (2014).

[36] Rodrigues, R. C., Berenguer-Murcia, Á., Carballares, D., Morellon-Sterling, R. & Fernandez-Lafuente, R. Stabilization of enzymes via immobilization: Multipoint covalent attachment and other stabilization strategies. *Biotechnol. Adv.* **52**, 107821. 10.1016/j.biotechadv.2021.107821 (2021). 34455028 10.1016/j.biotechadv.2021.107821

[37] Tian, Y. et al. Effects of protein concentration, pH, and NaCl concentration on the physicochemical, interfacial, and emulsifying properties of β-conglycinin. *Food Hydrocoll.* **118**, 106784. 10.1016/j.foodhyd.2021.106784 (2021).

[38] Sulyman, A. O., Igunnu, A. & Malomo, S. O. Isolation, purification and characterization of cellulase produced by *Aspergillus niger* cultured on *Arachis hypogaea* shells. *Heliyon* **6**, e05668. 10.1016/j.heliyon.2020.e05668 (2020). 33319112 10.1016/j.heliyon.2020.e05668 PMC7723808

[39] Gu, L. et al. Formulation of alginate/carrageenan microgels to encapsulate, protect and release immunoglobulins: Egg Yolk IgY. *Food Hydrocoll.* **112**, 106349. 10.1016/j.foodhyd.2020.106349 (2021).

[40] Jiang, Q., Li, S., Du, L., Liu, Y. & Meng, Z. Soft κ-carrageenan microgels stabilized pickering emulsion gels: Compact interfacial layer construction and particle-dominated emulsion gelation. *J. Colloid Interface Sci.* **602**, 822–833. 10.1016/j.jcis.2021.06.070 (2021). 34171747 10.1016/j.jcis.2021.06.070

[41] Chitra, R., Sathya, P., Selvasekarapandian, S. & Meyvel, S. Synthesis and characterization of iota-carrageenan biopolymer electrolyte with lithium perchlorate and succinonitrile (plasticizer). *Polym. Bull.* **77**, 1555–1579. 10.1007/s00289-019-02822-y (2020).

[42] Hong, T., Yin, J. Y., Nie, S. P. & Xie, M. Y. Applications of infrared spectroscopy in polysaccharide structural analysis: Progress, challenge and perspective. *Food Chem. X* **12**, 100168. 10.1016/j.fochx.2021.100168 (2021). 34877528 10.1016/j.fochx.2021.100168 PMC8633561

[43] Deng, W., Xu, Q., Hu, X. & Sheng, L. Structure and properties of egg white protein films modified by high-intensity ultrasound: An effective strategy. *Food Res. Int.* **157**, 111264. 10.1016/j.foodres.2022.111264 (2022). 35761576 10.1016/j.foodres.2022.111264

[44] Nandiyanto, A. B. D., Oktiani, R. & Ragadhita, R. How to read and interpret FTIR spectroscope of organic material. *Ind. J. Sci. Technol.* **4**, 97–118 (2019).

[45] Zdarta, J., Jędrzak, A., Klapiszewski, L. & Jesionowski, T. Immobilization of cellulase on a functional inorganic–organic hybrid support: Stability and kinetic study. *Catalysts* **7**, 374. 10.3390/catal7120374 (2017).

[46] Muley, A. B., Thorat, A. S., Singhal, R. S. & Babu, K. H. A tri-enzyme co-immobilized magnetic complex: Process details, kinetics, thermodynamics and applications. *Int. J. Biol. Macromol.* **15**(118), 1781–1795. 10.1016/j.ijbiomac.2018.07.022 (2018). 10.1016/j.ijbiomac.2018.07.022 30003912

[47] Gadaka, M. A. et al. Effects of immobilization on the kinetic parameters of partially purified cellulase from termites (*Macrotermes bellicosus*). *Acta Sci. Pharm.* **2**(6), 20–28 (2021).

[48] Romo-Sánchez, S., Camacho, C., Ramirez, H. L. & Arévalo-Villena, M. Immobilization of commercial cellulase and xylanase by different methods using two polymeric supports. *Adv. Biosci. Biotechnol.* **5**, 517–526. 10.4236/abb.2014.56062 (2014).

[49] El-Hadi, A. A., Kamel, Z., Hammad, A., Abu El-Nour, S. & Anwar, S. Optimization of *aspergillus hortai* cellulase immobilized on poly (Acrylamide-Co-Acrylic acid) for hydroxylation of cellulose rice straw wastes. *Glob. J. Pharma* **8**(4), 681–687 (2014). https://api.semanticscholar.org/CorpusID:46178744

[50] Tamilanban, R., Rajadas, S. E., Sounderrajan, V. & Harshavardhan, S. Immobilization of thermostable, bacterial cellulase from *Stenotrophomonas maltophilia* in agar-agarose matrices and its characterization. *Int. J. Biosci.* **13**(3), 198–208. 10.12692/ijb/13.3.198-208 (2018).

[51] Talekar, S. et al. Carrier free co-immobilization of alpha amylase, glucoamylase and pullulanase as combined cross-linked enzyme aggregates (combi-CLEAs): A tri-enzyme biocatalyst with one pot starch hydrolytic activity. *Bioresour. Technol.* **1**(147), 269–275. 10.1016/j.biortech.2013.08.035 (2013). 10.1016/j.biortech.2013.08.035 23999260

[52] Riordan, J. F. The role of metals in enzyme activity. *Ann. Clin. Lab. Sci.* **7**, 119–129 (1977). 192123

[53] Ishida, N., Okubo, A., Kawai, H., Yamazaki, S. & Toda, S. Interaction of amino acids with transition metal ions in solution (I) solution structure of L-lysine with Co(II) and Cu(II) ions as studied by nuclear magnetic resonance spectroscopy. *Agric. Biol. Chem.* **44**, 263–270. 10.1080/00021369.1980.10863934 (1980).

[54] Ladole, M. R., Mevada, J. S. & Pandit, A. B. Ultrasonic hyperactivation of cellulase immobilized on magnetic nanoparticles. *Bioresour. Technol.* **1**(239). 10.1016/j.biortech.2017.04.096 (2017). 117 – 26. 10.1016/j.biortech.2017.04.096 28501684

[55] Dal Magro, L., Hertz, P. F., Fernandez-Lafuente, R., Klein, M. P. & Rodrigues, R. C. Preparation and characterization of a Combi-CLEAs from pectinases and cellulases: A potential biocatalyst for grape juice clarification. *RSC Adv.* **6**(32), 27242–27251. 10.1039/C6RA03940E (2016).

[56] Muley, A. B., Chaudhari, S. A., Mulchandani, K. H. & Singhal, R. S. Extraction and characterization of chitosan from prawn shell waste and its conjugation with cutinase for enhanced thermo-stability. *Int. J. Biol. Macromol.* **111**, 1047–1058. 10.1016/j.ijbiomac.2018.01.115 (2018). 29366886 10.1016/j.ijbiomac.2018.01.115

[57] Dutta, K., Krishnamoorthy, H. & Dasu, V. V. Novel cutinase from *Pseudomonas cepacia* NRRL B 2320: Purification, characterization and identification of cutinase encoding genes. *J. Gen. Appl. Microbiol.* **59**, 171–184. 10.2323/jgam.59.171 (2013). 23863287 10.2323/jgam.59.171

[58] Elnashar, M. M. M. & Yassin, M. Covalent immobilization of β-galactosidase on carrageenan coated with chitosan. *J. Appl. Polym. Sci.* **114**, 17–24. 10.1002/app.30535 (2009).

[59] Abdel-Sater, M. A., Hussein, N. A., Fetyan, N. A. & Gad, S. A. Immobilization of cellulases produced by *Penicillium brevicompactum* AUMC 10987, using cross-linkage, chitosan-coating and encapsulation. *Catrina* **18**(1), 139–149. 10.21608/cat.2019.28624 (2019).

[60] Sarcina, R. et al. Immobilization of two endoglucanases from different sources. *Int. J. Environ. Agric. Biotechnol.* **2**(4), 1809–1813. 10.22161/ijeab/2.4.44 (2017).

[61] Ozyilmaz, G. & Gunay, E. Clarification of apple, grape and pear juices by co-immobilized amylase, pectinase and cellulase. *Food Chem.* **398**, 133900. 10.1016/j.foodchem.2022.133900 (2023). 35986994 10.1016/j.foodchem.2022.133900

[62] Chang, T., Siddiq, M., Sinha, N. K. & Cash, J. N. Commercial pectinases and the yield and quality of Stanley plum juice. *J. Food Process. Preserv.* **19**, 89–101. 10.1111/j.1745-4549.1995.tb00280.x (1995).

